# WORK PRECARITY AND THE AGING WORKFORCE: TRENDS IN HEALTH DISPARITY AMONG OLDER SERVICE SECTOR WORKERS

**DOI:** 10.1093/geroni/igad104.2356

**Published:** 2023-12-21

**Authors:** Renada Goldberg, Janette Dill, Jiyeon Kim

**Affiliations:** Simmons University, Boston, Massachusetts, United States; University of Minnesota, Minneapolis, Minnesota, United States; University of North Carolina at Chapel Hill, Chapel Hill, North Carolina, United States

## Abstract

Work precarity may affect workers’ physical, mental, and social well-being as navigating uncertain and insecure work conditions interacts with factors such as housing, family caretaking, and personal relationships. In particular, the service sector has long been characterized by work precarity as compared to other industries in the US, where noncollege workers are far more vulnerable to non-standard and low-wage work and Black, Indigenous, and workers of color are overrepresented. For older adults, structural and systemic inequities may exacerbate health conditions and accelerates biological aging. In this study, we measure work precarity among noncollege older workers (55-75 years old) in three large and growing service sector industries in the US: health care, retail, and food service, to access the impact of work precarity on older low-wage worker’s health outcomes, exits from the formal wage labor workforce, and linkages between work precarity and health disability.

